# The revised 2024 McDonald criteria can solve the misdiagnosis problem in multiple sclerosis: Commentary

**DOI:** 10.1177/13524585251396271

**Published:** 2025-12-13

**Authors:** Anneke van der Walt

**Affiliations:** Department of Neuroscience, School for Translational Medicine, Monash University, Melbourne, VIC, Australia Multiple Sclerosis and Neuroimmunology Service, Alfred Health, Melbourne, VIC, Australia

The 2024 revision of the McDonald diagnostic criteria^1^ for multiple sclerosis (MS) represents the most ambitious effort yet to harmonise early diagnosis with diagnostic accuracy. But can it solve the long-standing issue of MS misdiagnosis,^2^ which is more often a problem of misapplication than a failure of better-defined criteria?

Proponents highlight several landmark advances. Most striking is the paradigm shift away from dissemination in time (DIT) as an essential requirement. Historically, patients often waited for new lesions or a second clinical attack before diagnosis, which often delayed initiation of treatment. The 2024 criteria instead lean on a biologically grounded framework, allowing diagnosis at first presentation when characteristic lesions in four or five topographies are present, or when other highly specific markers are detected. By de-emphasising DIT, the new criteria reduce reliance on a tool that is both blunt and variable.

At the same time, the criteria incorporate objective markers of MS biology, particularly through neuroimaging.^3^ The central vein sign (CVS) and paramagnetic rim lesions (PRLs) offer impressive specificity: five or more CVS lesions confer 98% specificity, while a single PRL has been shown to have 100% specificity in real-world studies. Along with structured guidance for atypical presentations and age-specific safeguards, this may help counter overdiagnosis, particularly in challenging contexts such as radiologically isolated syndrome (RIS), paediatric cases or older adults with comorbidities.

However, critics caution that new tools do not fix old habits. Misdiagnosis in MS is less about flawed criteria and more about their misapplication – particularly overreliance on nonspecific MRI findings in patients with atypical symptoms. Misdiagnosis rates remain stubbornly high, with studies reporting that 12%–30% of patients referred to MS centres are ultimately reclassified.^4^ A major concern lies in the implementation gap. CVS and PRLs require imaging protocols and interpretative expertise that are not universally available. Similarly, optic nerve assessments with visual evoked potentials and optical coherence tomography require specialised equipment and standardisation – posing challenges in lower-resource settings.^5^

Despite this, the criteria explicitly allow flexibility: not every patient needs every test. PRLs and CVS are *not required* for diagnosis but are particularly helpful in ambiguous cases. The criteria also embed the principle of ‘no better explanation’ more deeply, with specific recommendations to test for MOG antibodies in children under 12 or to apply additional criteria in older adults with vascular comorbidities. It should be noted that much of the validation data for the revised criteria is drawn from cohorts in North America and Europe, which limits their generalisability across diverse populations.^4^

Ultimately, the 2024 McDonald criteria are not a panacea. They represent a decisive step towards biologically anchored, earlier diagnosis – but they will succeed only if coupled with careful clinical reasoning, education and humility. Without this, even the best criteria risk being applied too broadly, too quickly. Misdiagnosis will not be solved by criteria alone, but by how thoughtfully we use them.
